# Two families with quadrupedalism, mental retardation, no speech, and infantile hypotonia (Uner Tan Syndrome Type-II); a novel theory for the evolutionary emergence of human bipedalism

**DOI:** 10.3389/fnins.2014.00084

**Published:** 2014-04-22

**Authors:** Uner Tan

**Affiliations:** Department of Physiology, Medical School, Cukurova UniversityAdana, Turkey

**Keywords:** Uner Tan syndrome, ataxia, quadrupedalism, evolution, complex systems, self-organization

## Abstract

Two consanguineous families with Uner Tan Syndrome (UTS) were analyzed in relation to self-organizing processes in complex systems, and the evolutionary emergence of human bipedalism. The cases had the key symptoms of previously reported cases of UTS, such as quadrupedalism, mental retardation, and dysarthric or no speech, but the new cases also exhibited infantile hypotonia and are designated UTS Type-II. There were 10 siblings in Branch I and 12 siblings in Branch II. Of these, there were seven cases exhibiting habitual quadrupedal locomotion (QL): four deceased and three living. The infantile hypotonia in the surviving cases gradually disappeared over a period of years, so that they could sit by about 10 years, crawl on hands and knees by about 12 years. They began walking on all fours around 14 years, habitually using QL. Neurological examinations showed normal tonus in their arms and legs, no Babinski sign, brisk tendon reflexes especially in the legs, and mild tremor. The patients could not walk in a straight line, but (except in one case) could stand up and maintain upright posture with truncal ataxia. Cerebello-vermial hypoplasia and mild gyral simplification were noted in their MRIs. The results of the genetic analysis were inconclusive: no genetic code could be identified as the triggering factor for the syndrome in these families. Instead, the extremely low socio-economic status of the patients was thought to play a role in the emergence of UTS, possibly by epigenetically changing the brain structure and function, with a consequent selection of ancestral neural networks for QL during locomotor development. It was suggested that UTS may be regarded as one of the unpredictable outcomes of self-organization within a complex system. It was also noted that the prominent feature of this syndrome, the diagonal-sequence habitual QL, generated an interference between ipsilateral hands and feet, as in non-human primates. It was suggested that this may have been the triggering factor for the attractor state “bipedal locomotion” (BL), which had visual and manual benefits for our ape-like ancestors, and therefore enhancing their chances for survival, with consequent developments in the psychomotor domain of humans. This was put forward as a novel theory of the evolution of BL in human beings.

## Introduction

Locomotion on all fours can normally be seen in human infants during the crawling period. A typical infantile quadrupedalism involves the hand and knees alternating diagonally between left-hand, right-knee and right-hand, left-knee: diagonal sequence crawling on hands and knees. Hands and feet may also be used during this period (bear crawling: diagonal sequence crawling on hands and feet). Trettien (1900) reported on 150 children, 50% of whom exhibited diagonal crawling on hand and knees, 20% lateral crawling on hands and knees, and 9% diagonal crawling on hands and feet. Hrdlicka (1931) reported some cases of bear crawling in healthy children.

Nearly 100 years later from the first quadruped man discovered by Childs (1917), a consanguineous family with 19 siblings, 6 of them exhibiting a novel syndrome with habitual QL, mental retardation and impaired speech was reported, and their condition was named *Uner Tan syndrome* (Tan, 2005, 2006a,b,c; see Tan, 2010; Tan et al., 2012 for reviews).

Among cerebellar ataxias, UTS is a unique syndrome with substantial differences from other balance disorders such as disequilibrium syndrome (DES), Cayman ataxia, and Joubert syndrome (see Tan, 2010; Tan et al., 2012). In this context, Guertin (2013) emphasized that UTS is a *“recently identified and uniquely different neurological disorder.”* Genetic studies showed UTS to be a unique, genetically heterogeneous syndrome without infantile hypotonia (Ozcelik et al., 2008; Gulsuner et al., 2011).

The primary aim of the present work was to evaluate the members of two closely related novel families with UTS, residing in a small village near Diyarbakir, South-Eastern Turkey. Some of the siblings in these families exhibited infantile hypotonia along with the usual UTS symptoms: no speech, severe mental retardation, and late-onset quadrupedal locomotion (QL). These symptoms constitute UTS Type-II, and UTS without infantile hypotonia is now designated UTS Type-I. In addition, this study will consider ipsilateral limb interference during QL, and its possible role in the evolutionary emergence of human bipedalism.

The most prominent feature of UTS, which is diagonal sequence QL in which the hind limb touchdowns are followed by the contralateral forelimb touchdowns, is characteristic of most primates, while lateral-sequence QL, with a hind limb touchdown followed by an ipsilateral forelimb touchdown, is characteristic of most non-primate species (Hildebrand, 1967; Prost, 1969; Rose, 1973; Rollinson and Martin, 1981; Schmitt and Lemelin, 2002). However, diagonal-sequence QL has a locomotor disadvantage in that there is often interference between the ipsilateral limbs, i.e., the ipsilateral hands and feet may clash during QL, constraining the movement of the coincident ipsilateral limbs (Schmitt and Lemelin, 2002). In this context, Larson (1998) reported that *“a diagonal sequence/diagonal couplet walking gait creates a strong potential for interference between the ipsilateral hind and forelimbs.”*

Interestingly, the first fish-like tetrapods also exhibited diagonal-sequence QL nearly 400 MYA, indicating that this type of QL is phylogenetically the oldest locomotor trait, and has been preserved for millions of years (see Tan et al., 2012). Human beings use essentially the same ancestral neural networks, ancient locomotor traits such as the central pattern generators (see Guertin, 2013; Ivanenko et al., 2013) generating diagonal sequence QL during bipedal locomotion (BL) (Donker et al., 2001; Zehr et al., 2009), even though human beings have the most complex brains of all species, along with unique psychomotor actions (see Tan et al., 2012). In this context, Bem et al. (2003) reported: “*our findings support the hypothesis of a phylogenetic conservatism of the spinal locomotor networks generating axial motor patterns from agnathans to amphibians.”* Dominici et al. (2011) also highlighted the evolutionary conservation of the ancestral neural networks in several animal species, beginning with common primitives (see Stuart, 2007).

Although diagonal-sequence QL was the first to appear during locomotor evolution, it was coupled with a locomotor disadvantage. Namely, the hindlimb and ipsilateral forelimb touchdowns coincided, increasing the probability of collision between the two limbs (Hildebrand, 1968). This was also observed in all of the UTS cases. Animals may have used two strategies to avoid the ipsilateral interference between the fore and hindlimbs: (i) by altering limb angular positioning, and (ii) by altering footfall patterns so that the ipsilateral forelimb is not maximally retracted at the moment of hindlimb touchdown (Young, 2012). Apart from this overstriding of one limb against another, one more mechanism could be developed to overcome the ipsilateral limb interference, and that is a transition from diagonal-sequence to lateral-sequence QL during evolution, with the consequent emergence of animals with lateral-sequence QL. It seems that most of the non-human primates succeeded in striding one limb over the other to avoid the collisions, and many non-primate animals succeeded in the transition from diagonal-sequence QL to lateral-sequence QL.

Several theories have been put forward to explain the evolutionary emergence of human bipedalism, e.g., the fighting hypothesis (Carrier, 2011), energetics (Sockol et al., 2007), the carry hypothesis (Videan and McGrew, 2002), and aquatic ape theory (de Sarre, 1988). Although the first hominins with BL emerged about 7 MYA, the mechanisms by which our BL evolved remains unknown (Sockol et al., 2007). These theories actually seems to be concerned with the consequences of the evolutionary emergence of human bipedalism rather than the mechanisms of that evolution. Consequently, the second aim of this work was to evaluate a novel theory to explain the evolutionary emergence of the human bipedalism, comparing the diagonal sequence QL in UTS cases. The working hypothesis was that the human bipedalism might be an attractor state triggered by the motor coincidence between the ipsilateral limbs during diagonal-sequence QL in our ancestors.

## Methods

Cognitive abilities were evaluated using the mini mental state examination (MMSE) test, standardized for uneducated Turkish adults, and also known as the “Folstein Test” (Folstein et al., 1975). This test consists of a 30-point questionnaire measuring the individual's orientation, attention, calculation, recall, language and motor skills. The total possible score is 30 points, with equal or greater than 25 points being normal, 21–24 points indicating mild mental impairment, 10–20 points moderate, and 9 or below severe mental retardation (Mungas, 1991).

Brain MRI scans were obtained to visualize the cerebro-cerebellar structures of the patients' brains. The brains were scanned with a Siemens (Erlangen, Germany) 1.5 T scanner. A healthy individual from the same family was also subjected to the same procedure as a control. The brain MRI scans were evaluated by a specialist neuroradiologist. The physical characteristics of the cases, such as the head circumference, body height and weight were also measured. A written informed consent for scientific study of the families was obtained from the healthy fathers and mothers of the families, Branch I and II, respectively. The study was approved by the local ethics committee of Cukurova University.

According to the reports of the parents, no QL had been observed in previous generations, and the affected children were the results of the consanguineous marriages in Branch I and II families. We collaborated with Dr. Gleeson's laboratory at the University of California San Diego (USA) for the genetic analysis to be performed on healthy subjects, including fathers and mothers, as well as the affected individuals from the Branch I and Branch II families. Using the Qiagen reagents, DNA was extracted from the blood samples and a 5K SNP scan was performed, the results being analyzed using easy-linkage-Plus software (see Ali et al., 2012).

The videos of the cases were taken with permission of the families and patients, and saved as Figshare files, which can be downloaded using the citations for the videos. One can watch the videos by clicking Ctrl+ on the video address.

## Results

### Environment

**Figure 1:**
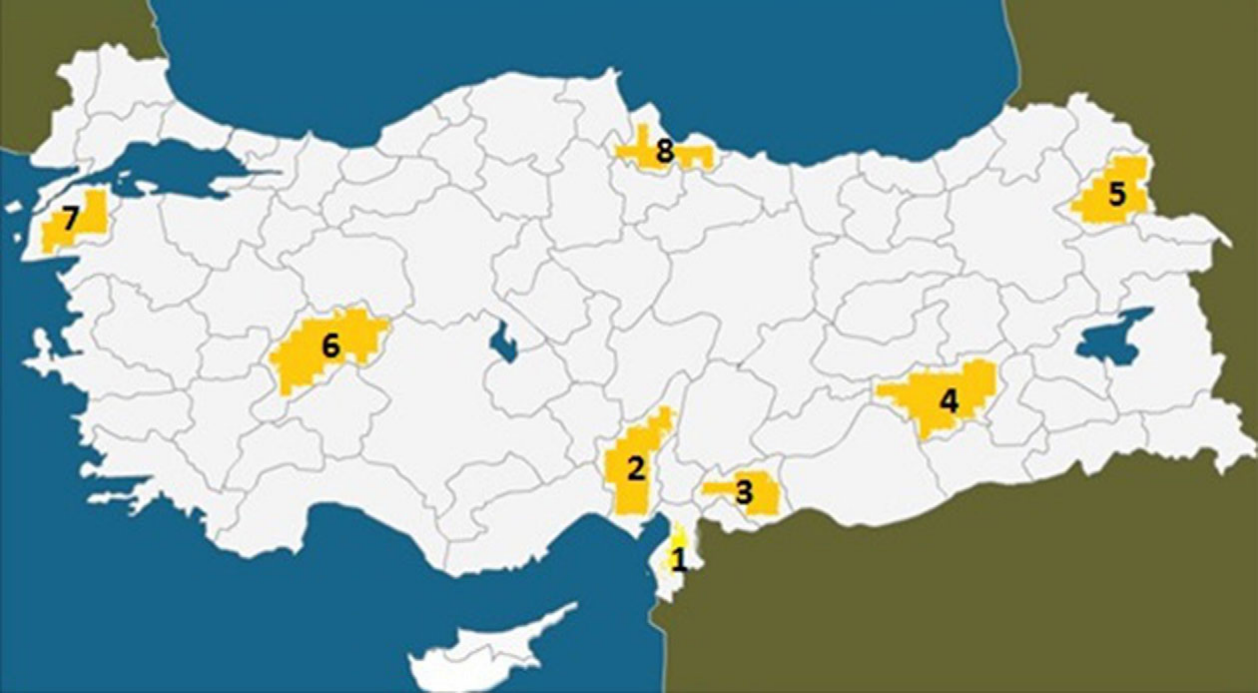
**Map of Turkey showing the places where the UTS cases were hitherto found between 1917 and 2012. (1)** Hatay (Iskenderun), 6 cases; **(2)** Adana, 3 cases; **(3)** Gaziantep, 4 cases; **(4)** Diyarbakir, 7 cases; **(5)** Kars, 2 cases; **(6)** Afyon, 3 cases; **(7)** Canakkale, 4 cases; **(8)** Samsun, 1 case, discovered in 1917.

There was one large and extremely poor core family with two closely related subfamilies, all residing in a small village with exclusively Kurdish inhabitants, located near Diyarbakir in South-Eastern Turkey (Figure 1, **area 4**). This was a poorly developed village with no school, water supply, or post office, but there was electricity. The families with affected members lived in small houses in the village, which had 62 houses and 3200 inhabitants, most of whom raised farm animals. The locations where previous UTS cases were found in Turkey are illustrated in Figure 3 (see also Tan et al., 2012).

### Genealogy

**Figure 2:**
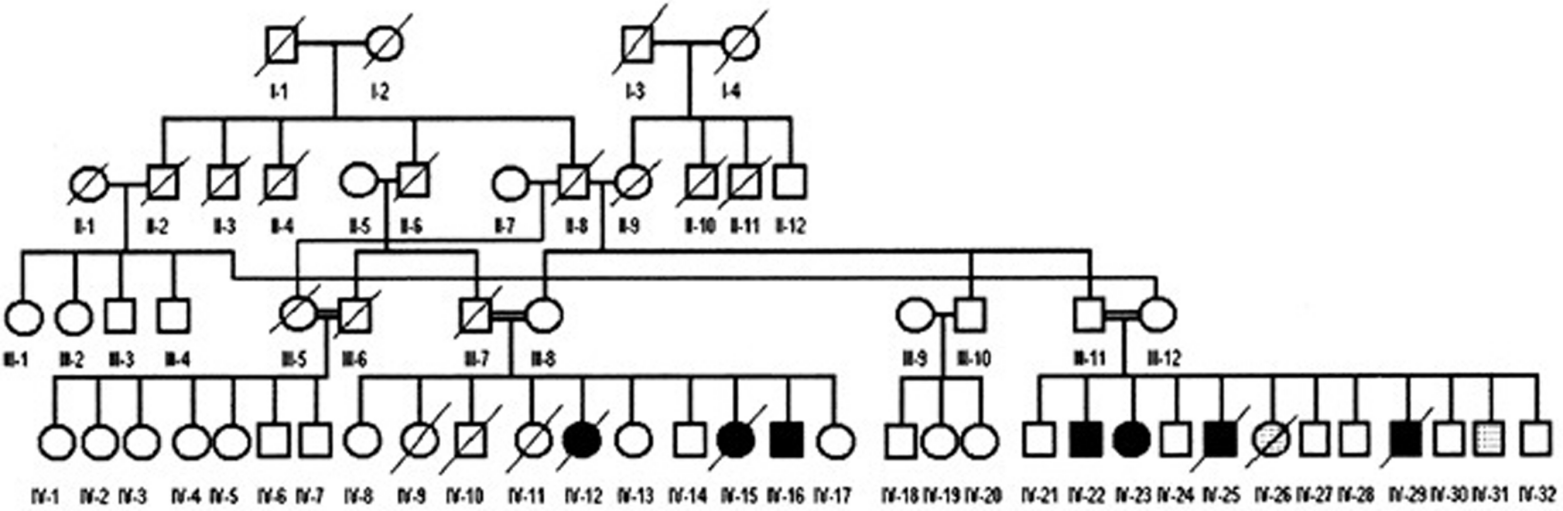
**Genealogic tree of the Diyarbakir family.** Squares: males, circles: females; filled symbols: cases affected with UTS; symbols filled with dots: bedridden cases. Double horizontal lines represent consanguineous marriages; diagonal lines represent deceased persons; roman numerals: generations; Arabic numerals: birth order of siblings. A specific combination of numerals, like IV-16, uniquely identifies each person.

The pedigree of the two related subfamilies is presented in Figure 2. The mother (III-8, 50 years) and father (III-7, deceased at 57 years) of the first branch were first cousins (II-6 and II-8). There were 10 siblings in Branch II (IV-8 to IV-17: six normal (IV-8 to IV-10; IV-13, IV-14 and IV-17), two deceased daughters (IV-12 and IV-15) both with UTS, who died of unknown causes at the age of 2 and 9 years respectively, and one living son with UTS (IV-16). Another sister (IV-11) had severe hypotonia and was bedridden and died at the age of about 2 years of unknown causes. The siblings of the second family Branch II also had consanguineous parents (III-11 and III-12) who were children of cousins (II-2 and II-8). There were 12 siblings in this family, six of them being healthy (IV-21, IV-24, IV-27, IV-28, IV-30, IV-32), and six affected with various degrees of psychomotor disorders; of these, IV-26 and IV-31 were bedridden.

### Branch I, case IV-16

**Figure 3:**
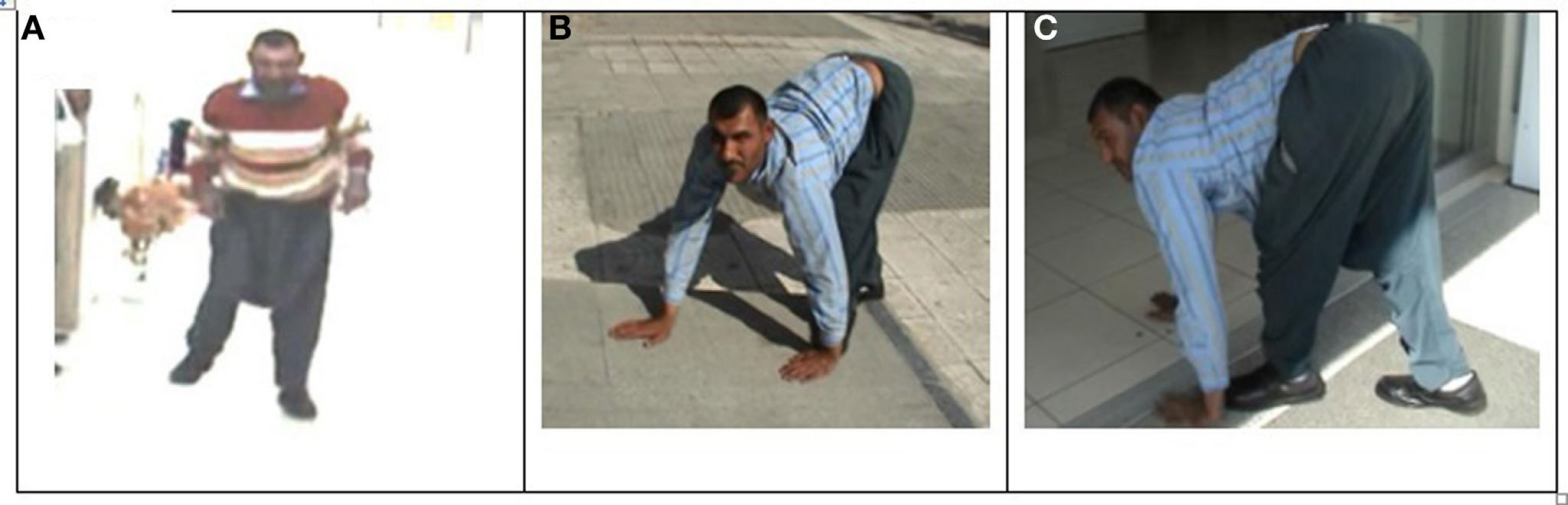
**Case IV-16: ataxic upright walking (A), diagonal QL with interference between left hand and left foot (B,C)**.

The patient designated IV-16 (28 years, male) and belonging to the Branch I family, was first encountered begging on the street, walking on all four extremities and exhibiting diagonal sequence QL. He had not been referred to a hospital at that time. After permission was granted by himself and his parents, neurological examinations and MRI scanning were carried out and a blood sample was taken for genetic analysis. Figure 3 illustrates his ataxic lateral-sequence BL Figure 3A and diagonal-sequence QL Figures 3B,C), with ipsilateral arm to leg and hand to foot interference on the left side. The gestation of this patient had been without complication, and he had been delivered at term with the help of a neighbor's wife. According to his mother, he had been hypotonic, like a jelly, just after birth, but suckling was well developed. He started to speak with dysarthria at 7 years, could sit up without help at the age of 10 years, crawled on hands and knees at 12 years, and started to walk on hands and feet at 16 years. He could easily stand up now (at 28 years) without assistance, walking upright forwards and backwards, but with truncal ataxia and his legs apart more than 25–30 cm (see Video 1: IV-16 BL). His infantile hypotonia had progressively disappeared over the years. Now, he is a strong, lively, and friendly man, joking very often, and not complaining about his obligatory QL (see Video 2: IV-16 QL). He did not go to school and is still illiterate.

The patient was able to speak using just a few 100 words necessary for everyday living, despite a mild dysarthria. He could construct only simple sentences, without using “and,” and “with.” In the Mini-Mental-State Examination test standardized for uneducated Turkish adults, the patient could not correctly answer the questions pertaining to the year, season, time, or country, and he could not understand the word “city.” He could recall three words and three items, could name the objects, repeat a short sentence, fold a paper, construct a sentence related to his home, could imitate the mimics, but could not draw a watch or name the days backwards. In total, he had 18 items correct from 30 items. To assess the patient's cognitive abilities further, the Wechler Adult Intelligence Scale-Revised (WAIS-R) (1981) was used. The verbal, performance, and full scale IQ scores were found to be 53, 50, and 46, respectively.

**Figure 4:**
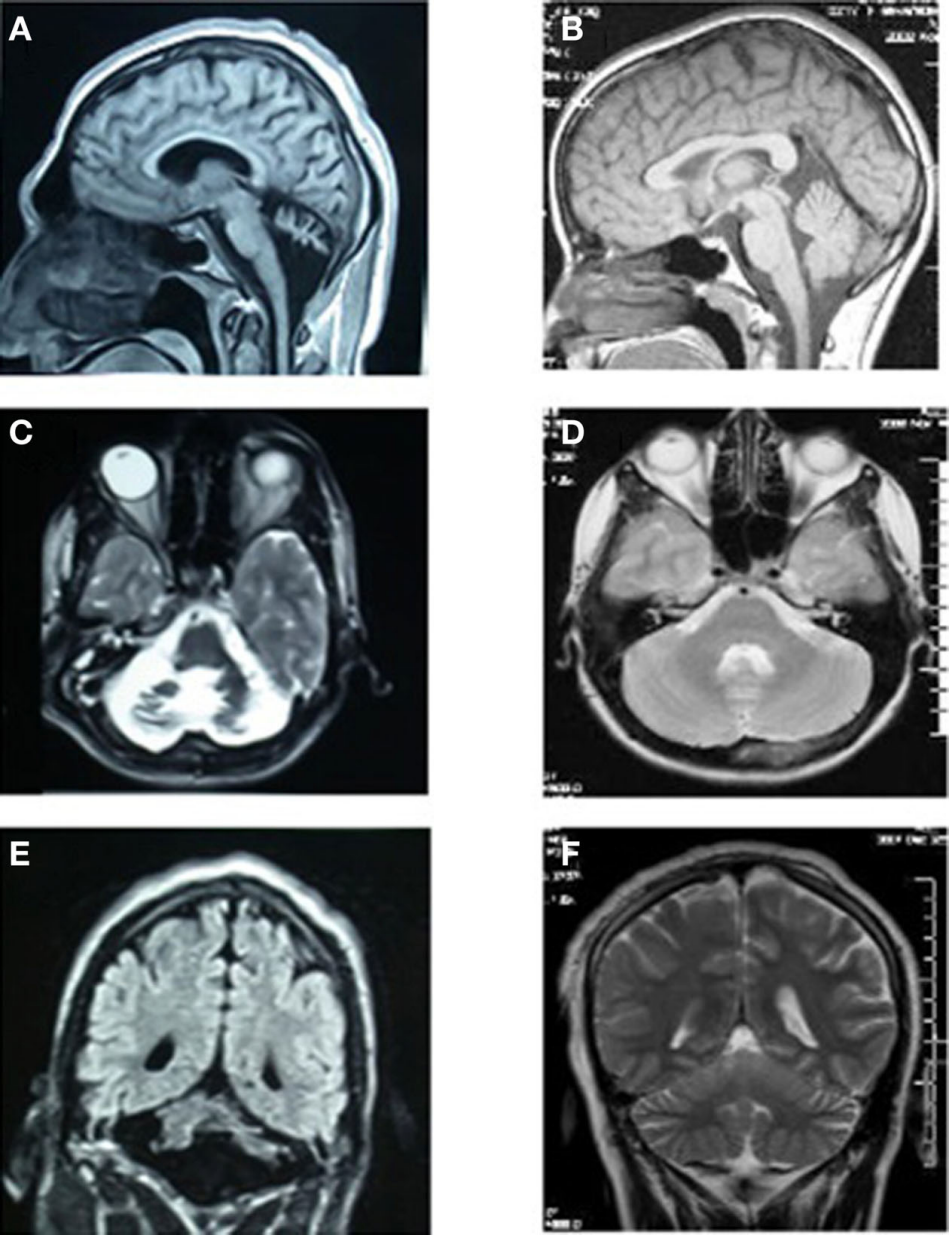
**MRI scans of the patient IV-16 (left) and the unaffected relative (right). (A,B):** midsagittal; **(C,D)**: axial; **(E,F)**: coronal sections.

MRI scans of the midsagittal, axial, and coronal sections of the brains of patient IV-16 and his unaffected brother are depicted in Figure 4. The midsagittal MRI scan showed a prominent cerebello-vermial hypoplasia, a thin corpus callosum, and gyral simplification, especially in the frontal cortex (Figure 4A) compared to the healthy subject, who had normal anterior, posterior superior, and posterior inferior vermial lobes, and well developed cortical gyral structures (Figure 4B). In the axial MRI section, there is severe cerebellar hypoplasia with just visible vermis (Figure 4C), compared to the control subject's well-developed cerebellum and cerebellar vermis (Figure 4D). In the coronal section, the severe cerebello-vermial hypoplasia were visible in the patient (Figure 4E) compared to the control subject, who had a well-developed cerebellum and cerebellar vermis (Figure 4F). Otherwise, the basal ganglia, thalamus, bulbus, and hippocampus seemed to be normal and similar to the control subject, but the pons appeared to be mildly hypoplastic.

### Branch II

The fathers of the mother (45 years) and father (55 years) of the second branch (III-11, III-12) were their respective uncles (II-3 and II-8). There were 12 siblings in this family, six of them being healthy (IV-21, IV-24, IV-27, IV-28, IV-30, IV-32) and the remaining six being affected with varying degrees of psychomotor disabilities (IV-22, 23, 25, 26, 29, 31). The cases IV-22 (25 years) and IV-23 (20 years) were a man and woman with fully developed UTS exhibiting habitual QL with severe mental retardation, and no speech except a few nonsense sounds. The main physical and neurological characteristics of the family members were as follows:

The mother (III-12) was quite normal with no impaired speech or intelligence; head circumference (50 cm) and height (154 cm) were within normal ranges for the Turkish population.

The father (III-11) was also normal with unimpaired intelligence and speech. Head circumference (52 cm) and height (155 cm) were within normal ranges. However, the deep tendon reflexes (DTR) were hypoactive in the lower extremities, which exhibited muscular atrophy.

Case IV-21 (30 years, male) did not show any psychomotor abnormalities, including locomotion and cognitive faculties.

Case IV-22 (25 years, male) showed fully developed UTS with truncal ataxia, habitual QL, severe mental retardation, and no speech except a few sounds. The patient could easily stand up, but he needed the help of a wall or a person for support. He fell down if he tried to make a step (Video 3: IV-22). There was neither nystagmus nor tremor. In the upper extremities, muscle tonus and muscle strength were well-developed with normal DTRs. In the lower extremities, muscle strength was normal, but muscle tonus was hypertonic, and the DTRs were hyperactive, brisk (grade 3). The muscles below the knees were atrophic. The Babinski sign was bilateral positive.

**Figure 5:**
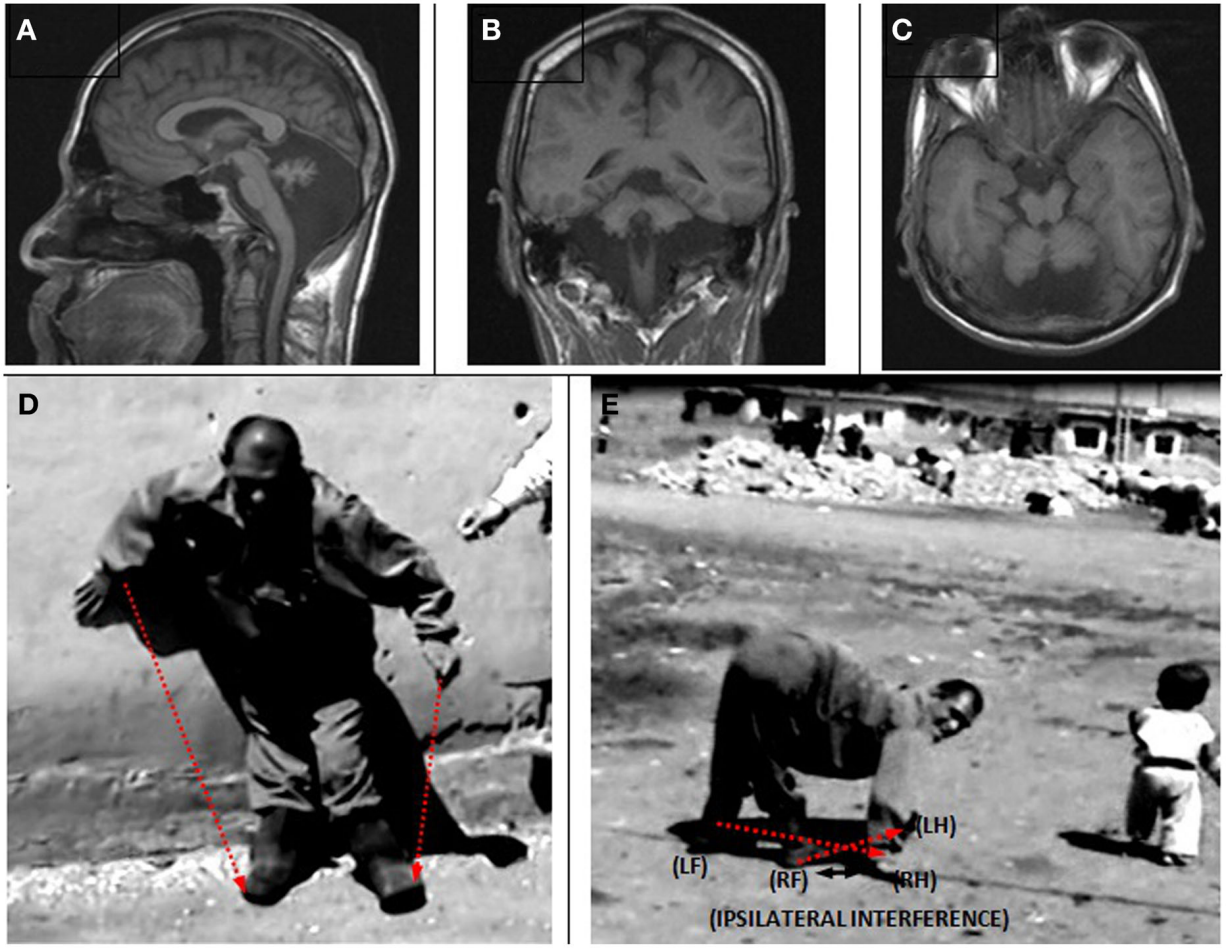
**Case IV-22. Sagittal (A), coronal (B) and axial (C) MRI scans. (D)** Releasing from the wall to make a step: notice the parallel action of the left arm and left leg; **(E)** diagonal-sequence QL soon after releasing from the wall: notice the interference between the right arm and right leg.

The MRIs depicted in Figure 5A (sagittal), Figure 5B (coronal), and Figure 5C (axial) show a diffuse cerebello-vermial hypoplasia, evidenced by prominent cerebellar foliae, in addition to mild cortical gyral simplifications. The basal ganglia, thalamus, bulbus, hippocampus and pons seemed unimpaired. Figure 5D shows the subject swinging the left arm parallel with the left leg as he attempted to make a step from the wall supporting him without falling down due to truncal ataxia. When he fell down after releasing from the wall, he immediately started to walk on all four extremities, exhibiting diagonal-sequence QL (Figure 5E; Video 3: IV-22).

**Figure 6:**
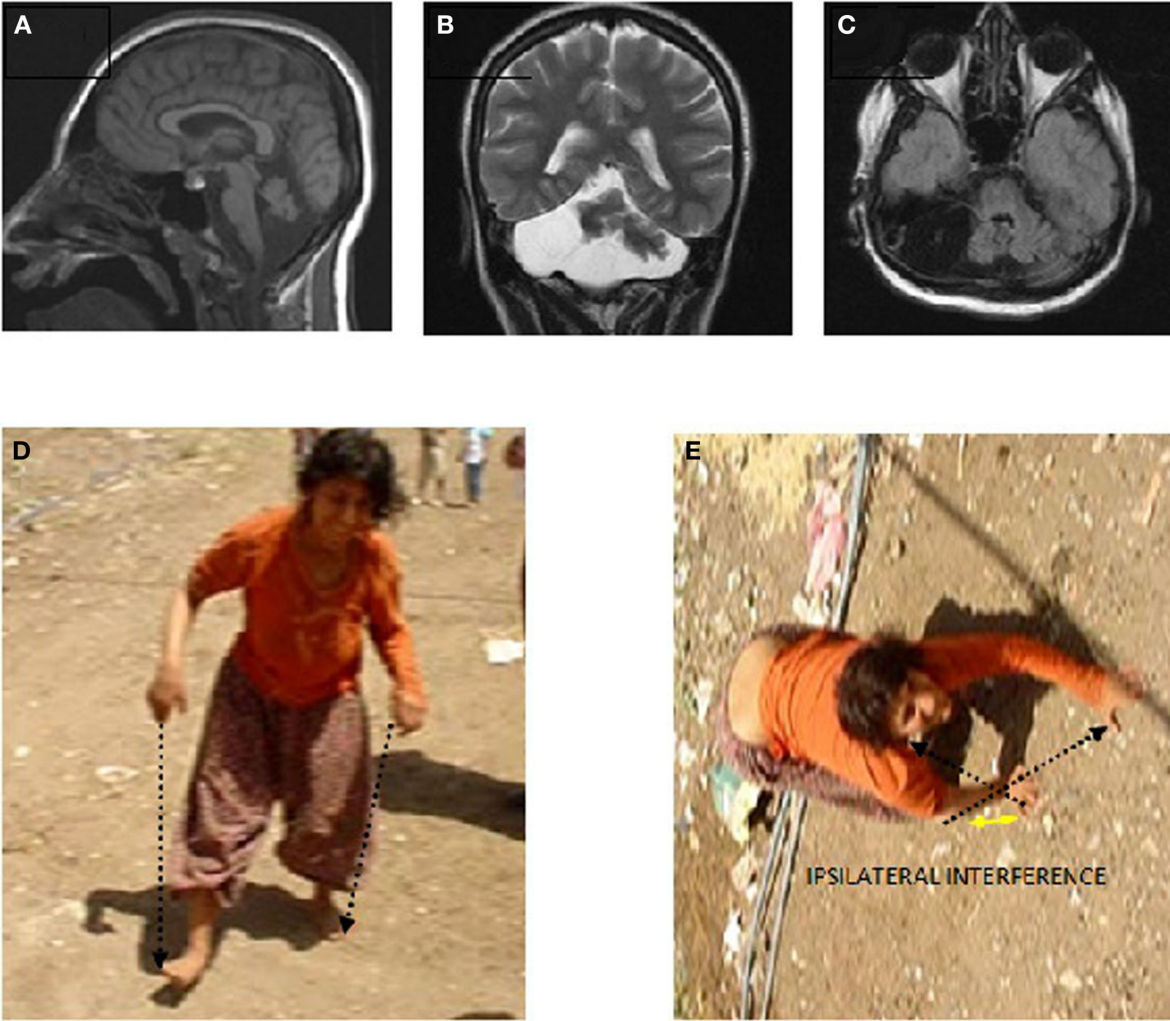
**Case IV-23. Sagittal (A), coronal (B), and axial (C) MRI scans. (D)** Parallel-sequence ataxic BL: right arm-right leg and left arm-left leg (see arrows); **(E)** diagonal-sequence QL: left arm-right leg and right arm-left leg (see arrows).

Case IV-23 (20 years, female) exhibited UTS, with upwards nystagmus, bilateral dysmetria, dysdiadochokinesia, atrophy in the muscles of the legs below the knees, no plantar reflex, normal DTRs in the upper extremities, hyperactive DTRs in the lower extremities, severe mental retardation, and no speech except a few nonsense sounds. Her MRI scan showed cerebello-vermial hypoplasia (Figure 6A: sagittal; Figure 6B: coronal, and Figure 6C: axial sections). The basal ganglia, thalamus, bulbus, hippocampus, and pons seemed unimpaired.

She could easily stand up and walk back and forth, but with difficulty due to the truncal ataxia. Figure 6 depicts her upright, parallel sequence (right arm-right leg vs. left arm-left leg) BL Figure 6D, and diagonal-sequence (right arm-left leg vs. left arm-right leg) QL Figure 6E. Video 4 IV-23 shows the locomotor functions of this patient: standing up, walking upright, BL with truncal ataxia, and walking on all fours without truncal ataxia.

**Figure 7:**
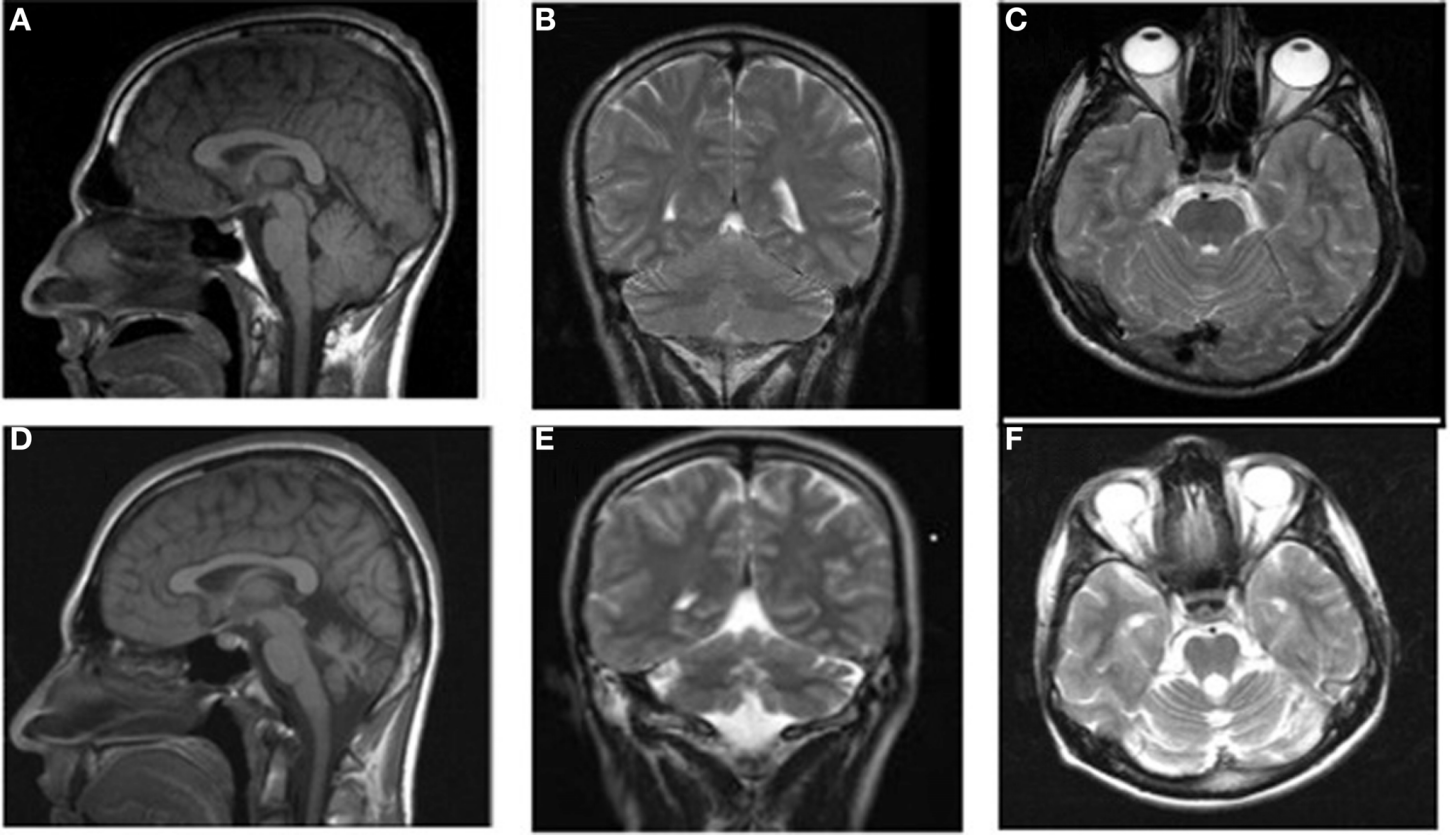
**MRI scans from a healthy subject: sagittal (A), coronal (B), and axial (C) and the patient IV-24: sagittal (D), coronal (E), and axial (F)**.

**Figure 8:**
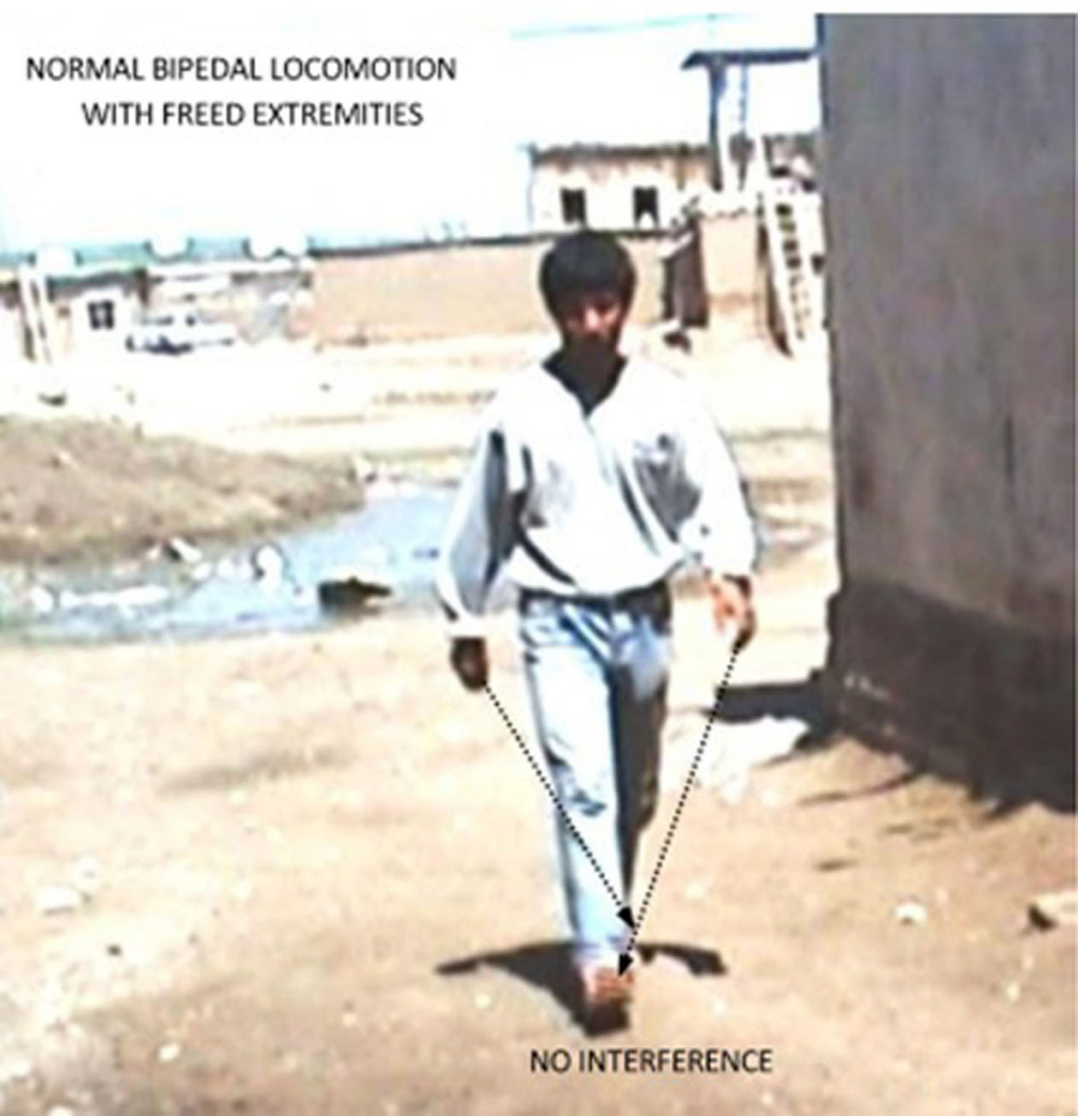
**Diagonal-sequence BL in the case IV-24: left arm-right leg and right arm-left leg as indicated by the arrows.** Notice no interference between arms and legs with freed hands and feet.

Case IV-24 (18 years, male) exhibited well-balanced bipedal walking and running (Video 5). The Babinski sign was absent, DTRs were normal in the upper extremities but hyperactive in the lower extremities. His MRI showed a mild cerebello-vermial hypoplasia, without gyral simplification in the cerebral cortex (Figure 7). The basal ganglia in thalamus, bulbus, hippocampus and pons seemed to be unimpaired in this case. He had a mild mental retardation with limited vocabulary, and was attending the school for handicapped children in Diyarbakir. Figure 8 illustrates the diagonal sequence (right leg-left arm vs. left leg-right arm) BL of this case.

Case IV-25 (deceased at 10 years) had UTS with QL, mental retardation, and no speech.

Case IV-26 (deceased at 15 years) had no ambulation, was hypotonic, bedridden, with no speech except a few sounds (no UTS).

Cases IV-27, (15 years, male), IV-28 (10 years, male), and IV-32 (1 years, male) had no neurological signs or symptoms; speech and intelligence were not impaired.

Case IV-29 (deceased at 7 years) had UTS Type-II with quadrupedalism, mental retardation, childhood hypotonia, and no speech.

**Figure 9:**
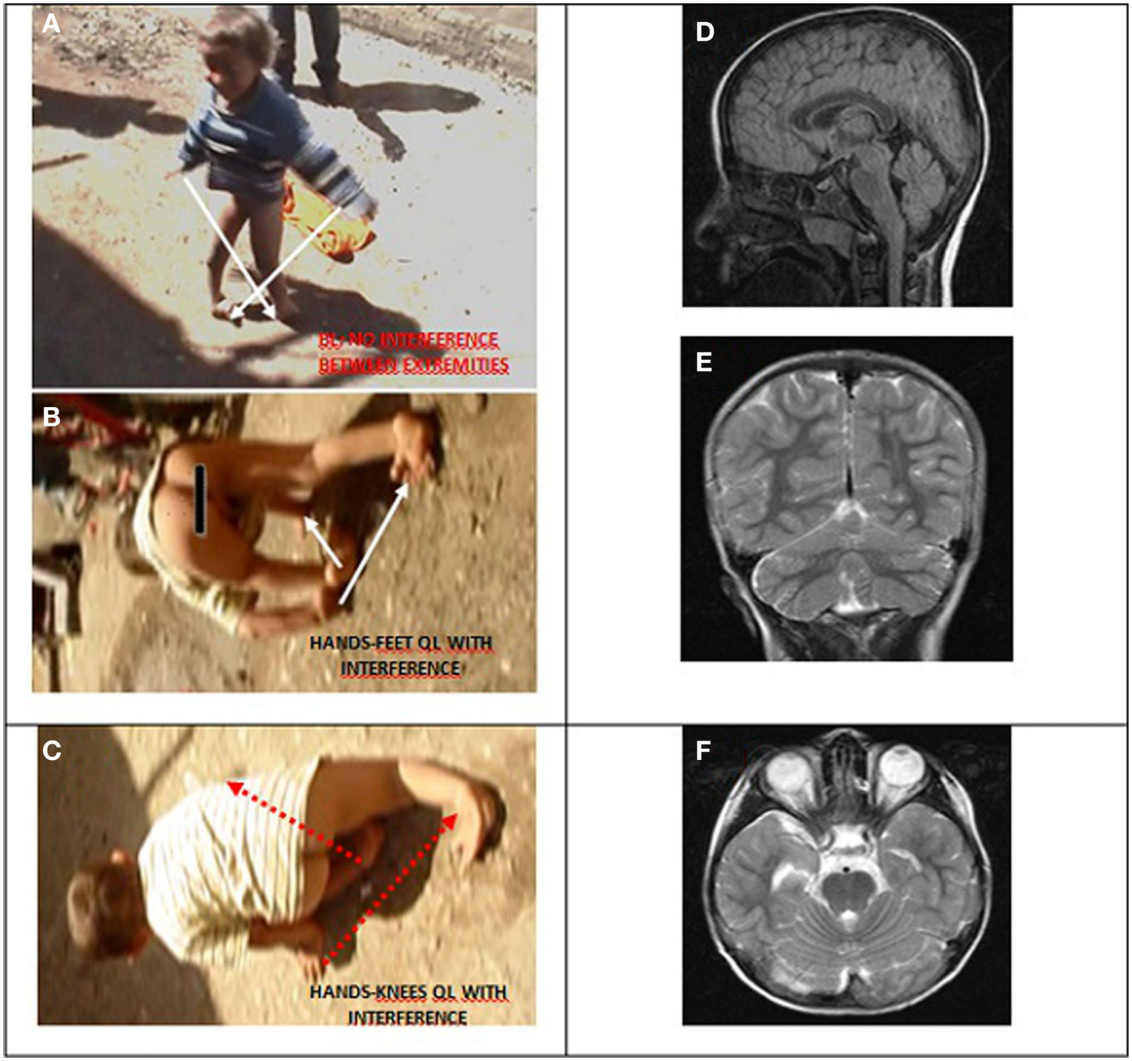
**Case IV-30, 5 years old male with congenital talipes equinovarus in both feet. (A)** Diagonal-sequence BL: right arm-left leg and left arm-right leg (see arrows). **(B)** Diagonal-sequence QL on hands and feet: left arm-right leg and right arm-left leg (see arrows). Notice the interference between the ipsilateral extremities on the left side; **(C)** diagonal sequence QL on hands and knees. Midsagittal **(D)**, coronal **(E)**, and axial **(F)** MRI scans.

Case IV-30 (5 years, male) had congenital talipes equinovarus in both feet, which were turned inward and could not easily be moved into the normal position. Locomotion was facultative: he could easily stand up and walk and run on two feet, but he walked on hands and knees for slow actions and used QL for fast actions. He could understand, but not speak except for producing a few unintelligible sounds. His cognitive status could not be assessed because of difficulties in communication. Otherwise, he seemed to be a bright but naughty child. Figure 9 depicts his diagonal-sequence BL Figure 9A, QL on hands and feet Figure 9B, and on hands and knees Figure 9C, and the midsagittal Figure 9D, coronal Figure 9E, and axial Figure 9F MRI scans. No cerebello-vermial hypoplasia or any other anomalies in the cerebral structures were visible in these MRI scans. Video 6 CASE IV-30 (ALL GAITS) shows the fast quadrupedal running on hands and club feet and slow QL on hands and knees, followed by upright BL on club feet.

Case IV-31 (3 years, male) was severely hypotonic, could not hold his head up, had horizontal nystagmus, severe muscular atrophy in the muscles of the upper and lower extremities. DTRs could not be elicited. Bilateral Babinski was negative. He was bedridden.

### Genetic analysis

The genealogy presented in Figure 2 suggested an autosomal recessive transmission from consanguineous parents. As a result of our collaboration with the Gleeson Lab at the University of California, San Diego, a possible mutation in the VLDLR (very low density lipoprotein receptor) gene was looked for and excluded in the affected cases of the Branch I and II families. In this context, the VLDLR gene involved in neuroblast migration in the cerebral cortex and cerebellum showed a missense mutation in members with UTS in two closely related families, as first mentioned by Ozcelik et al. (2008).

The results of the analysis for Single Nucleotide Polymorphism (5K-SNP) and the whole genome-wide approach were inconclusive: linkage plots showed areas where some homozygocities were found, but the linkage peaks did not reach statistically significant levels.

The clinical findings of the affected members of the families are presented in Table 1.

**Table 1:** **Clinical characteristics of the UTS Type-II and other cases**.

**Variables**	**Case IV-16**	**Case IV-22**	**Case IV-23**	**Case IV-24**	**Case IV-30**	**Case IV-31**
Sex	Male	Male	Female	Male	Male	Male
Age	28 years	25 years	20 years	18 years	5 years	3 years
Gait	QL	QL	QL	BL	Facultative	No
Ataxia	Yes	Yes	Yes	No	No	Bedridden
Standing	Normal	Normal	Normal	Normal	Normal	No
BL	Ataxic	No	Ataxic	Normal	Normal	No
Muscle tone	Normal (5/5)	Normal (5/5)	Normal (5/5)	Normal (5/5)	Normal (5/5)	Hypotonia (0/5)
Early hypotonia	Yes	Yes	Yes	No	No	Yes
DTR: upp. ext.	3+++ (hyper)	2++ (normal)	2++ (normal)	2++ (normal)	Unknown	0 (Absent)
DTR: low. ext.	3+++ (hyper)	3+++ (hyper)	3+++ (hyper)	3+++ (hyper)	Unknown	0 (Absent)
Cerebellar hypopl asia	Yes	Yes	Yes	Mild	No	Unknown
Head size	Normal	Normal	Normal	Normal	Normal	Normal
Speech	Dysarthric	No	No	Normal	Delayed	No
Mental retardation	Mild	Severe	Severe	Mild	No	Unknown
Babinski	Absent	Present	Absent	Absent	Unknown	Absent
Nystagmus	Horizontal	No	Upward	Absent	Unknown	Horizontal
Tremor	Mild	No	No	Absent	Unknown	No
MMSE	22/30	3/30	2/30	21/30	No reply	No reply

Upp. ext., upper extremity; low. ext., lower extremity; BL, bipedal locomotion.

## Discussion

The characteristics of a novel variant of UTS was presented in two closely related families: UTS Type-II, which included childhood hypotonia without ambulation. The infantile hypotonia slowly disappeared and was replaced with normal muscle tone and QL during adolescence. In the previously described UTS Type-I cases, there was no childhood hypotonia, but the major symptoms, such as the consistent QL, mental retardation and dysarthric or no speech, were the same (for reviews see Tan, 2010; Tan et al., 2012).

The early phase of UTS Type-II, with severe hypotonia, no ambulation, truncal ataxia, severe psychomotor retardation, no speech, and a marked cerebellar hypoplasia, may be related to the long-known DES (Hagberg et al., 1972) and its clinical synonym Cayman ataxia (Brown et al., 1984; Nystuen et al., 1996). In contrast to these non-progressive cerebellar ataxias, in the UTS Type-II cases, the severe muscular hypotonia gradually disappeared and was replaced by strong muscles appropriate for walking, at least on all four extremities. The DES, Cayman ataxia and Joubert syndromes are mainly characterized by lifelong hypotonia with no ambulation even in adulthood.

Like UTS Type-I, UTS Type-II is also a rare cerebellar ataxia syndrome. This is consistent with the unpredictability of the outcomes of dynamical complex systems with a strong tendency to self-organize (Guarini and Onofri, 1993; Gribble, 2001). The extremely slow locomotor development in the UTS Type-II cases may be due to the slow progression of the self-organized, adaptive developmental processes (Tan, 2010; Tan et al., 2012; Karaca et al., 2013). The nature of UTS as a developmental disorder was recently dealt with in detail in a book chapter (Karaca et al., 2013).

In all of the UTS Type-II cases, the deep tendon reflexes were normal in the upper extremities but hyperactive in the lower extremities, except case IV-16 who had hyperactive DTRs in both upper and lower extremities. These results suggest that these cases may be affected by the upper motoneuronal lesions in addition to the cerebellar disorders, since the hyperactive stretch reflexes are generally elicited if the cortico-spinal pathways are interrupted at supraspinal levels (Walker, 1990).

With regard to VLDLR gene mutation in some cerebellar ataxias, Moheb et al. (2008) argued that *“VLDLR deficiency alone is sufficient to cause the human DES phenotype.”* Although a similar mutation in the same gene was also found in some families with UTS Type-I (Caglayan, 2008), this was not the rule either for DES or UTS cases. For instance, no VLDLR mutation could be detected in the UTS Type-II families in the present work. Similarly, Melberg et al. (2011) reported that their cases diagnosed as DES were negative for mutations in the VLDLR gene. Thus, DES may not always be associated with a mutation in the VLDLR gene.

The UTS cases showed genetic heterogeneity (see Karaca et al., 2013): VLDLR mutation in the Antep and Canakkale families (Ozcelik et al., 2008), CA8 in the Iraqi family (Turkmen et al., 2009), WDR81 in the Iskenderun family (Gulsuner, 2011; Gulsuner et al., 2011), and ATP8A2 in Adana family (Onat et al., 2013). Thus, no single gene defect may explain the emergence of UTS. This weakens the arguments about the genetic origins of UTS, and is consistent with findings on the minor genetic influence on the well-known syndromes. Accordingly, Maher (2008) argued that *“even when dozens of genes have been linked to a trait, both the individual and cumulative effects are surprisingly small and nowhere near enough to explain earlier estimates of heritability.”* Moreover, it is well known that similar mutations of a gene may be associated with different expressions of the same phenotype, i.e., *“similar genetic lesions can have entirely different phenotypes”* (Prasun et al., 2007).

Considering the above mentioned Mahler's argument about the small effect of genes in heritability, it is likely that genetics may play only a minor role in the origins of UTS, but the inconclusive results of the whole genome analysis in this work make a definitive conclusion impossible. However, it may be concluded that additional agents would probably contribute to the emergence of this syndrome. Accordingly, Hall (2011) argued *“as the past 70 years made abundantly clear, genes do not control development. Genes themselves are controlled in many ways, some by modifications of DNA sequences, others by external and/or environmental factors.”* There is one factor shared by all of the UTS families: the low socio-economic status, with associated low income, under-nutrition, illiteracy, and parental neglect. Thus, the unfavorable socio-economic status gains importance for the emergence of the whole spectrum of cerebro-cerebellar signs and symptoms in families with UTS, and affects the genetic expression (see Karaca et al., 2013). The association with the epigenetic status was highest in the most socio-economically deprived group of individuals and lowest in the least deprived group (McGuinness et al., 2012). The epigenetic status (DNA-methylation patterns) was also affected by dietary factors, even during embryogenesis, with further consequences in adult life (Mathers et al., 2010; Thompson et al., 2010; Thornburg et al., 2010). Similarly, the UTS Type-II families living under extremely low socio-economic conditions may also have been subjected to epigenetic changes during embryogenesis (see also Tan, 2010; Tan et al., 2012; Karaca et al., 2013). These epigenetic changes may be subjected to developmental disorders under the influence of self-disorganization. The rareness of these conditions may be due to the unpredictability of the outcomes of the complex systems, in which self-organization and self-disorganization occur. This may also be the reason why UTS was discovered only in families with low socio-economic status, the syndrome being so rare and not occurring in most low socio-economic status populations. That is, the low socio-economic status with its unfavorable effects on the prenatal development through epigenetic mechanisms may exert its effects within the brain, which is a dynamic system with highly complex information networks tending to self-organize attractor states, which are unpredictable and uncontrollable (Heylighen, 2008), like the emergence of UTS with impairments in upright posture and cognitive functioning resulting in ancestral QL, mental retardation, and dysarthric or no speech. In addition to unpredictability of the outcome of a complex system with self-organizing properties, the inbreeding may also be considered as an additional factor in rareness of the syndrome, i.e., a combination of poverty and inbreeding.

The cases with diagonal-sequence QL showed an ipsilateral extremity interference, constraining the movement of the coincident ipsilateral hands and feet. There is no interference between the ipsilateral extremities in tetrapods with lateral-sequence QL. Although the ipsilateral limb coincidence may be disadvantageous, this may have had important evolutionary consequences with regard to the emergence of BL in human beings. Hominids with ancestral BL would have had a better developed cortico-cerebellar system. In agreement with this, the affected UTS cases showed an impaired cortico-cerebellar motor control. In this context, Smaers et al. (2011) accentuated the importance of the cortico-cerebellar connections in human evolution: *“neural systems involving profuse cortico-cerebellar connections are a major factor in explaining the evolution of anthropoid brain evolution.”* Moreover, a functionally better developed brain may also induce better conscious control over any sustained perturbation in the locomotor system (Malone and Bastian, 2010), to overcome, for instance, the disadvantageous locomotor effect of the ipsilateral limb interference during QL, by the emergence of a new locomotor attractor state, human bipedalism. This may occur in a complex system by adaptive self-organization occurring within the brain of our ancestors, without any external selection process, in line with Waldrop (1990), who noted: *“the tendency of complex dynamical systems to fall into an ordered state without any selection process whatsoever.”* Oudeyer (2006) also reported: *“the explanation of the origins of forms and structures in the living can not only rely on the principle of natural selection, which should be complemented by the understanding of physical mechanisms of form generation in which self-organization plays a central role.”* Malone and Bastian (2010) reported on the role of conscious control of locomotor adaptation to a sustained perturbation. Our ancestors cannot be excluded from such a conscious control of locomotion. Namely, consciously perceiving the benefits of BL, such as freeing the hands for fine manipulations, foraging or carrying items, reaching food in previously inaccessible environments, for carrying babies to provide better protection. The more beneficial attractor state, upright posture with BL, would then emerge during evolution of the locomotor system. These benefits would enhance their chances of survival, creating habitually upright-walking human beings, with consequent developments in the psychomotor domain.

The strong potential for the interference between the ipsilateral hind and forelimbs during diagonal-sequence QL may have been the triggering factor for our ancestors to try to overcome this locomotor disadvantage by standing up, using BL. This would then result in more accurate specialization of the fore and hindlimbs: feet only for walking and running; hands only for manual actions such as grasping, throwing, handling, exploring, and tool-making. This is the novel theory for the evolutionary emergence of bipedalism in human beings, suggested for the first time in the present work: the ipsilateral limb interference theory for the evolution of human bipedalism.

The child, Case IV-30, with congenital club feet, exhibited all forms of locomotion: BL, QL on hands and feet, and crawling on hands and knees. He preferred upright BL and QL for fast actions: he always ran rapidly on two feet (BL) or on all fours (QL) despite the club feet, but he crawled on hands and knees for slow actions. He seemed to be a bright child with normal brain structures seen in MRI scans. We had previously described similar cases without psychomotor disorders: 4- and 12-year-old bright males, who also showed facultative locomotion: BL for slow and QL for fast actions (Tan and Tan, 2009). So, an impaired brain is not necessary for the emergence of QL (see also Karaca et al., 2012), in contrast to the argument that human QL should be considered as an epiphenomenon caused by neuro-developmental malformation and ataxia (Hertz et al., 2008). It was, instead, suggested that human quadrupedalism may spontaneously emerge as the outcome of the self-organizing brain processes in human beings with entirely normal brains, by taking advantage of the ancestral neural networks preserved for about 400 million years since the first appearance of fish-like tetrapods (Karaca et al., 2012). The 5-year-old child (Case IV-30) apparently utilized all possibilities for locomotion, including the most recent neural networks for BL, and the ancestral neural networks for QL, which will probably be suppressed later on and replaced by the most recently emerged BL in human beings.

## Conclusions

Two closely related consanguineous families with siblings exhibiting UTS were presented. They lived in a small village near Diyarbakir, in South-Eastern Turkey. The affected siblings had infantile hypotonia, which had gradually disappeared by adolescence, and was replaced by QL during adulthood (UTS Type-II: a novel variant of UTS). No mutation was found in the VLDLR gene, but the genetic analysis was inconclusive. No single gene had so far been identified as a single factor directly involved in the emergence of UTS; consanguinity was not a prerequisite for the emergence of the syndrome, consistent with the Editorial in Nature Genetics: *“it is unlikely that consanguinity contributes significantly to polygenic and multifactorial disease once socio-economic variables have been controlled for.”* The only factor shared by all of the UTS families was their low socio-economic status, which may have led to epigenetic changes during embryogenesis, with postnatal psychomotor impairments extending from childhood to adulthood. The patients exhibited diagonal-sequence QL similar to non-human primates. The first appearing fish-like tetrapods during the Devonian period also used diagonal-sequence QL, nearly 400 MYA, suggesting that the neural networks, such as the spinal central pattern generators, for this kind of QL were preserved throughout the evolution of tetrapods, and human beings are still using the neural networks for diagonal-sequence QL. Non-primates with lateral-sequence QL did not show such an evolutionary course.

The diagonal-sequence QL was accompanied by interference between the ipsilateral hands and feet in patients, similar to that also found in non-human primates, which would be disadvantageous for effective locomotion. It was suggested that this locomotor disadvantage may have had evolutionarily advantageous consequences for human beings. Namely, the evolutionary development of human bipedalism may have been the result of long-lasting adaptive self-organizing processes to overcome the interference effect of quadrupedalism. The attractor state, bipedalism, could then be achieved, which would free the hands from locomotion, making them more suitable for skilled actions. This is the ipsilateral limb interference theory for the evolution of human bipedalism, put forward for the first time in the present work. This may be plausible if the evolution is considered as the outcome of self-organization with unpredictable attractor states, occurring not under the influence of previously established programs or single factors, such as neural and/or genetic codes (see also Tan, 2010). The beneficial effects of habitual BL would then enhance the chances for survival and further developments in the psychomotor domain of human beings.

## Conflict of interest statement

The author declares that the research was conducted in the absence of any commercial or financial relationships that could be construed as a potential conflict of interest.

## Supplementary material

The Supplementary Videos for this article can be found online at:

Video 1 **UNER TAN SYNDROME TYPE-II CASE IV-16a. figshare**. **http://dx.doi.org/10.6084/m9.figshare.813372**

Video 2 **UNER TAN SYNDROME TYPE-II CASE IV-16b. figshare**. **http://dx.doi.org/10.6084/m9.figshare.813379**

Video 3 **UNER TAN SYNDROME TYPE-II CASE IV-22. figshare**. **http://dx.doi.org/10.6084/m9.figshare.813380**

Video 4 **UNER TAN SYNDROME TYPE-II CASE IV-23. figshare**. **http://dx.doi.org/10.6084/m9.figshare.813381**

Video 5 **CASE IV-24. figshare**. **http://dx.doi.org/10.6084/m9.figshare.862920**

Video 6 **CASE IV-30. figshare**. **http://dx.doi.org/10.6084/m9.figshare.862940**
